# Current Aspects and Future Considerations of EGFR Inhibition in Locally Advanced and Recurrent Metastatic Squamous Cell Carcinoma of the Head and Neck

**DOI:** 10.3390/cancers13143545

**Published:** 2021-07-15

**Authors:** Bhamini Patel, Nabil F. Saba

**Affiliations:** 1 Department of Medicine, Emory University School of Medicine, Atlanta, GA 30322, USA; bppate5@emory.edu; 2 Department of Hematology and Medical Oncology, Winship Cancer Institute, Emory University, Atlanta, GA 30322, USA

**Keywords:** head and neck squamous cell carcinoma, EGFR inhibition in head and neck cancer, combination immunotherapy and EGFR in head and neck cancer, EGFR monoclonal antibodies, EGFR and chemotherapy in SCCHN, Epidermal Growth Factor and squamous cell carcinoma, pembrolizumab and monoclonal antibody in SCCHN

## Abstract

**Simple Summary:**

Squamous cell carcinoma of the head and neck (SCCHN) is a debilitating disease that affects hundreds of thousands of individuals worldwide and has a high mortality rate. Mainstay treatment largely consists of surgery, radiation, and chemotherapy which has been met with significant morbidity. The epidermal growth factor receptor is one that which plays a major role in cell signaling and has been extensively studied in locally advanced (LA) and recurrent metastatic (RM) SCCHN. This review paper details the major roles of the epidermal growth factor receptor (EGFR), previous and current EGFR inhibition therapeutics, resistance mechanisms, and the possible integration of immunotherapy and EGFR inhibition in this disease process.

**Abstract:**

Recurrent metastatic (RM) and locally advanced (LA) squamous cell carcinoma of the head and neck (SCCHN) are devasting disease states with limited therapeutic options and poor overall survival. Targeting the epidermal growth factor receptor (EGFR) is one area that has helped improve outcomes in this disease. Anti-EGFR based therapies have been shown to improve overall survival and mitigate the significant toxicities incurred from standard radiation, chemotherapy, and/or surgical options. Cetuximab, the most well-studied anti-EGFR monoclonal antibody, has demonstrated a positive impact on outcomes for RM and LA SCCHN. However, the development of early resistance to cetuximab highlights the need for a wider arsenal of therapy for RM and LA diseases. The use of immune checkpoint inhibitors has recently transformed the treatment of recurrent SCCHN. Drugs such as pembrolizumab and nivolumab have demonstrated success in recent clinical trials and have been approved for the treatment of advanced disease. Given the positive results of both EGFR targeted agents and immune checkpoint inhibitors, ongoing trials are studying their synergistic effects.

## 1. Introduction

Squamous cell carcinoma of the head and neck (SCCHN) is a devasting disease that accounts for over 600,000 new cancer cases worldwide on a yearly basis with nearly 45% of patients having regional lymph node metastasis at the time of diagnosis [1]. Despite advances in diagnostics and treatment of SCCHN, overall 5-year survival remains stagnant at only 50% with significant rates of second primaries [2]. The epidermal growth factor receptor (EGFR) plays an integral role in tumor biology and its expression has been correlated with more aggressive disease. It is well-known that EGFR is overexpressed in over 90% of SCCHN tumors, and close to 10–30% of SCCHN carcinomas demonstrate EGFR gene amplification [3,4]. The EGFR, also known as ErbB1 or Her-1, is a member of the complex receptor tyrosine kinase (RTK) family which also includes ErbB2 (Neu, Her-2), ErbB3 (Her-3), and ErbB4 (Her-4) [5,6].

EGFR is comprised of three components which include an extracellular component with four domains to assist in ligand binding, a transmembrane segment, and an intracellular component which contains the tyrosine kinase domain that facilitates downstream signaling cascades [3]. Numerous EGFR ligands exist, including epidermal growth factor, transforming growth factor-alpha, and heparin-binding EGFR, which bind to domains I and III of the extracellular EGFR. Ligand binding exposes domain II, promoting either homodimerization with other EGFR proteins or heterodimerization with other RTK family members. This dimerization induces autophosphorylation of the intracellular tyrosine residues and activates the eventual signaling cascades influencing gene expression, proliferation, apoptosis inhibition, metastasis, and cell mobility [3,5,7].

There are multiple mechanisms of EGFR activation. As described above, stabilization of receptor dimers through ligand binding serves as one of the three major processes. A second mechanism involves mutations in the receptor such as truncation of the N-terminal, which may allow for receptor stability and dimerization without ligand assistance. Lastly, the EGFR is a free-flowing complex within the lipid bilayer; when in close proximity to other EGFR complexes it can stimulate autophosphorylation and activate downstream signaling cascades. This ligand independent dimerization is extremely relevant in tumor cells where overexpression of the EGFR molecule can lead to increased activation, thereby promoting pathways for tumorigenesis [7,8]. 

## 2. Role of EGFR in SCCHN

**Figure 1:**
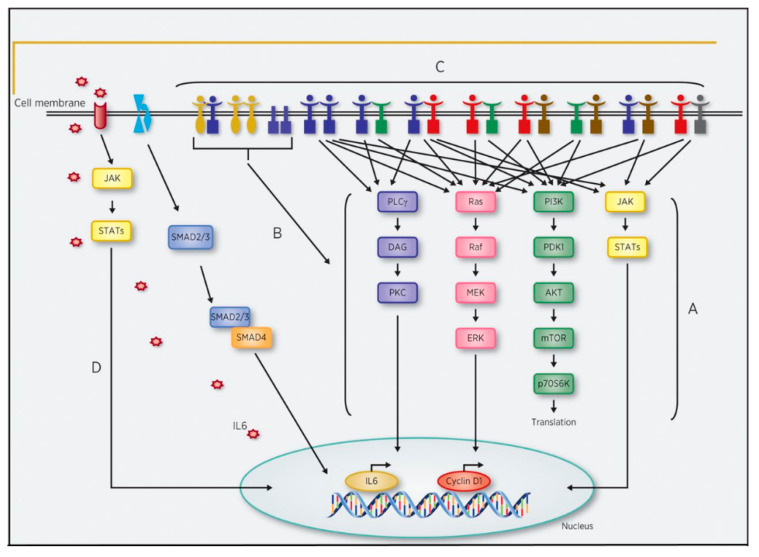
This figure represents potential resistance mechanisms to EGFR inhibition. (A) Activation mutations or amplifications of downstream EGFR pathways; (B) Overexpression of MET protooncogene and expression of EGFR variant III; (C) Heterodimerization between other RTK family members; (D) Activation of TGFβ-IL6 axis. Reused from: Targeting the EGFR and Immune Pathways in Squamous Cell Carcinoma of the Head and Neck (SCCHN): Forging a New Alliance [9].

The EGFR family receptors are found in various areas including epithelial, mesenchymal, and neuronal tissues. They serve to regulate critical aspects of cell physiology including cell survival, division, proliferation, and differentiation [3,9]. These vital regulatory actions are performed after ligand binding and activation through the Ras/MAPK pathway, PI3K/AKT pathway, the phospholipase C/protein kinase C cascade, and/or the signal transducers and activators of transcription (STAT) pathways, as noted in Figure 1 [10,11].

EGFR is capable of stimulating signaling pathways that can alter the cell cycle. Overexpression of this receptor in transgenic mice during organogenesis has proven lethal [6]. It has been postulated that high levels of EGFR in SCCHN may be related to poor prognosis and high expression of the receptor and its ligand may be associated with decreased disease-free survival (DFS) and overall survival (OS). However, unlike tyrosine kinase mutations occurring predominantly in non-small cell lung cancer (NSCLC), there is no known correlation between EGFR related biomarkers or specific activating mutations within the EGFR that could predict EGFR inhibitor efficacy in SCCHN [5,12]. 

EGFR signaling is heavily regulated through complex mechanisms at the plasma membrane and during receptor internalization. This regulation is impaired in oncogenic EGFR due to overexpression, which results in increased ligand-independent homo- and heterodimerization, or genetic mutations allowing activated EGFR to bypass endocytosis or lysosomes for degradation [11]. These changes in EGFR regulation result in tumorigenesis and create numerous obstacles that prevent effective treatment of SCCHN.

## 3. Targeting EGFR in SCCHN

Most SCCHN are locally advanced, stage III or stage IV diseases at the time of diagnosis. Treatment in this setting often consists of a multimodal approach with chemotherapy, radiation therapy (RT), and/or surgery. As expected, these treatment modalities are associated with significant toxicity and despite aggressive treatment, 5-year survival is only 50% [13]. In attempts to improve survival and maintain quality of life, several molecularly based therapies have been heavily investigated including monoclonal antibodies, small molecule tyrosine kinase inhibitors (TKIs), serine/threonine-specific protein kinase inhibitors, and cyclin-dependent kinase inhibitors [14]. We will focus this review on the molecular therapies that inhibit EGFR activity in SCCHN. 

Cetuximab, a chimeric IgG1 monoclonal antibody against EGFR, is a monoclonal antibody for the treatment of LA and RM SCCHN approved by the US Food and Drug Administration. The promising results of cetuximab therapy in SCCHN may be attributed to several possible anti-tumor mechanisms. Cetuximab serves as a competitive ligand that binds to the extracellular domain of the EGFR with higher affinity than its natural ligand, thus preventing subsequent interaction [2,7]. In addition to direct EGFR blockade, cetuximab also decreases EGFR expression via internalization and degradation of the receptor, thereby preventing further downstream cascade signaling [5,15]. Lastly, cetuximab’s IgG1 backbone can bind NK cells and activate antibody-dependent cellular cytotoxicity (ADCC), enabling immune cells to target and kill specific cells [15,16,17]. 

Other EGFR monoclonal antibodies such as panitumumab, zalutumumab and nimotuzumab have been investigated, but have failed to show similar survival advantages in SCCHN [15]. Although all are monoclonal antibodies, they do not possess identical mechanisms of inhibition. Panitumumab is an IgG2 mAb that inhibits ligand binding, but it does not possess the additional benefit of ADCC, which may explain the discrepancies observed in clinical outcome when compared to cetuximab [15]. In addition, panitumumab was introduced at a later date and patients on these clinical trials may have had access to cetuximab and other systemic agents to treat their metastatic disease. Both zalutumumab and nimotuzumab are IgG1 mAbs, but they have also failed to show similar advantages to cetuximab in OS [18]. 

Most small molecule tyrosine kinase inhibitors (TKIs) are competitive inhibitors of adenosine-5′-triphosphate (ATP) binding and can inhibit multiple oncogenic tyrosine kinases. They have been approved for several hematologic, lymphoid, and solid malignancies. However, there is a subset of TKIs which solely inhibit members of the RTK family. These include single-target TKIs, such as erlotinib and gefitinib in addition to multi-targeted TKIs such as lapatinib, afatinib, and dacomitinib [19]. These agents bind to the intracellular domain harboring the intrinsic tyrosine activity of the EGFR and prevent activation of pathways such as Ras/MAPK or PI3K/AKT [12,14]. The activity of these agents has been limited in SCCHN with responses below single agent cetuximab.

## 4. Resistance Mechanisms to EGFR Inhibition

As discussed, there are currently multiple investigational treatments underway to target EGFR. However, the high rates of recurrence and treatment failure need to be further addressed. One possible explanation can be attributed to the development of varying resistance patterns to SCCHN treatment modalities. Therefore, a deeper understanding of the development of anti-EGFR resistance patterns in SCCHN may hold the key to unlocking the potential of these agents. As a single agent, cetuximab has an overall response rate (ORR) of only 13% and a median time to progression of 70 days suggesting possible development of early drug resistance [20]. Proposed mechanisms of resistance to EGFR targeted therapies include increased ligand production, upregulation of EGFR expression, mutations in KRAS, BRAF, NRAS, and PIK3CA genes, expression of the EGFR truncation mutation *EGFRvIII*, or upregulation of other ErbB family members [21]. Figure 1 highlights some of these resistance mechanisms.

Studies have demonstrated overexpression of alternative members of the RTK family, notably ErbB2/HER2 and ErbB3/HER3, in SCCHN which confer treatment resistance by promoting growth and poor differentiation [22]. In preclinical studies, increased levels of ErbB2/HER2 were associated with treatment resistance to cetuximab in several different cancer lines including SCCHN cell lines [23]. In vitro analysis of SCCHN cell lines demonstrated a similar association with gefitinib, where the addition of an ErbB2 antibody improved gefitinib’s inhibitory effect [24]. It should be noted that almost 39% of tumor samples from patients with untreated SCCHN had overexpression of ErbB2/HER2, which suggests that it can serve as a possible target for therapy [16]. ErbB3/HER3 resistance may be mediated by its receptor ligand, HRG1, such that upon binding, HER3 heterodimerizes and is activated. Increased expression of HRG1 has been correlated with worse outcomes in SCCHN through activation of HER3 [25]. Several studies have reported elevated HRG1 levels in SCCHN compared to other solid tumors [26]. 

EGFR-independent signaling patterns can also confer resistance in SCCHN treatment. It is postulated that the PI3K signaling pathway can mediate cetuximab-resistance through mutation in the phosphatidylinositol-4,5- bisphosphate 3-kinase catalytic subunit alpha. In preclinical data, targeting this mutation with a PI3KA inhibitor and cetuximab inhibited growth in the cetuximab-resistant cell lines [27]. Studies also suggest the JAK/STAT pathway may mediate cetuximab resistance. STAT3 activation was elevated in patients who received cetuximab treatment compared to no treatment. Additionally, inhibiting STAT3 in cell lines with either intrinsic or acquired cetuximab resistance demonstrated decreased cell growth [28].

Altered expression in proteins involved in epithelial-to-mesenchymal transition (EMT) is a well-known mechanism for chemoresistance, tumor invasion and metastasis. Several studies have suggested its association with RTK resistance in SCCHN [29]. One study assessed the NGF/TrkA axis, which activates downstream signaling cascades such as Ras/MAPK, PI3K/AKT, and PLCy resulting in cell proliferation, invasion, and even metastasis. Overexpression of the NGF/TrkA axis conferred a STAT3 mediated resistance to erlotinib where inhibiting TrkA resulted in increased erlotinib sensitivity [30]. Erlotinib resistance was also demonstrated in SCCHN cell lines that expressed high levels of E-cadherin repressor delta-crystallin enhancer binding factor 1 (δEF1 or ZEB1). However, erlotinib sensitivity was regained after knockdown of δEF1, suggesting its potential use as a marker for RTK treatment response rates [16]. In another study, an EMT protein, cortactin, affected downstream signaling pathways inducing gefitinib resistance [31]. 

Additionally, EGFR activation induces cell cycle progression and can mediate cyclin D1 transcription or stabilization [10]. Cyclin-D1 in complex with CDK4/CDK6 augments cell cycle proliferation via phosphorylation and inactivation of the tumor suppressor retinoblastoma protein [10,32]. *CCND1* encodes for cyclin-D1 and preclinical data have linked EGFR inhibition resistance in SCCHN with the overexpression of *CCDN1* [33]. This interaction suggests CDK4/CDK6 inhibitors in conjunction with monoclonal antibodies such as cetuximab, may be of significance in SCCHN. Despite encouraging results reported in a single arm phase II trial of palbociclib and cetuximab, a recent confirmatory randomized phase II trial assessing palbociclib with cetuximab vs. single agent cetuximab in platinum-resistant, cetuximab-naïve SCCHN carcinoma failed to report a significant benefit in median OS or PFS [34,35].

## 5. Overcoming Resistance in EGFR Therapy in SCCHN

Given the numerous mechanisms for the development of treatment resistance against monoclonal antibodies and TKIs in SCCHN, it is crucial to find strategies to overcome these resistance patterns. Much of the data surrounding resistance patterns in anti-EGFR treatment modalities are currently under investigation. Some treatment modalities have reached phase II or III in development and aim to inhibit multiple aspects of the EGFR signaling pathway. These therapies can target other members of the RTK family through multitargeted TKIs or mAbs, inhibition of parallel pathways, or affect downstream signaling cascades. 

Lapatinib, afatinib, and dacomitinib are single agent TKIs that target both EGFR and other ErbB family receptors [36]. Lapatinib is a dual inhibitor of EGFR and HER2, and it has been studied in conjunction with chemoradiotherapy (CRT) in either LA or RM SCCHN. A phase II study in therapy-naïve LA SCCHN suggested lapatinib had an ORR of 17% when used prior to CRT [37]. However, more recent studies demonstrated no response, regardless of prior EGFR inhibition with stable disease as the best response [38]. A phase II study assessed capecitabine and lapatinib as first line therapies for RM SCCHN. Although the study met the primary endpoint of an OS of 9.3 months, the authors did not believe this response rate was due to lapatinib as only two patients overexpressed HER2, and progression free survival (PFS) curves matched previously reported data of capecitabine alone [39]. A phase III trial with adjuvant lapatinib and concurrent CRT followed by maintenance lapatinib in stage II to IVA SCCHN in high-risk surgically resected patients failed to show additional survival benefits and was associated with higher toxicity compared to placebo [40]. 

Afatinib is an irreversible inhibitor of EGFR, HER2, and HER4 thereby inhibiting most homo-heterodimerization of the ErbB family receptors. In preclinical data, afatinib has demonstrated a dose-dependent antiproliferative effect, a slight improvement of radiosensitivity in in-vitro cells, and significant tumor growth delay with daily administration [41]. It has shown comparable activity to cetuximab in RM SCCHN, with continued benefit after crossover suggesting minimal cross-resistance [42]. To further assess this the LUX-Head and Neck 2 study examined adjuvant afatinib after complete response from CRT and found no benefit in disease-free survival compared to placebo in patients with unresected, intermediate to high risk SCCHN [43]. Ongoing studies in LA SCCHN include dual inhibition such as afatinib and cetuximab or afatinib with pembrolizumab (Table 1). 

Dacomitinib is similar to afatinib and irreversibly binds to EGFR, HER2, and HER4 receptors. Preclinical studies not only demonstrated dacomitinib’s equal efficacy but also showed a reduction in EGFR activity and downstream Akt and ERK pathways compared to cetuximab and erlotinib [44]. Dacomitinib also demonstrated clinical activity as a monotherapy in RM SCCHN with a partial response in eight (13%) and stable disease in 36 (57%) patients [45]. Another phase II trial assessed dacomitinib in patients with progressive RM SCCHN on platinum-based chemotherapy and reported a partial response in 10 (21%) and stable disease in 31 (65%) patients [46]. There are currently no ongoing studies with dacomitinib. 

Inhibitors of other pathways in ongoing trials include PI3K inhibitors such as PX-866 and BYL719, with preliminary data suggesting a benefit from BYL719. Inhibition of PI3KCA gene mutation via copanlisib in conjunction with cetuximab is also being studied [36]. Another synergistic inhibitory combination involves anti-EGFR therapy and various inhibitors of the PI3K/Akt/mTOR pathway, including mTOR inhibitors temsirolimus and everolimus. Initial preclinical data suggested possible synergetic effects of mTOR and EGFR inhibition. However, phase II studies have failed to prove these theories in clinical practice. The combination of temsirolimus and cetuximab in cetuximab-resistant SCCHN showed poor PFS, but did demonstrate a clinically significant response rate of 3.6–9.1 months in 12.5% of patients [47]. A similar study showed no added benefit of everolimus and erlotinib in platinum-resistant SCCHN [48]. A phase Ib study assessing everolimus with cetuximab and carboplatin for RM SCCHN demonstrated an ORR of 61.5% (all partial responses) and a PFS of 8.15 months [49]. These results highlight the need for more studies with combination regimens.

## 6. Role of EGFR Inhibition in Definitive Therapy

Bonner et al conducted a landmark phase III clinical trial assessing cetuximab and RT vs. RT alone in patients with stage III or IV LA SCCHN and demonstrated a prolonged median duration of control (24.4 vs. 14.9 months), OS (49 vs. 29.3 months), and PFS in the cetuximab group [50]. Cetuximab monotherapy in patients with progressive disease on platinum-based therapy was found to be well-tolerated and had an ORR of 13%, a disease control rate of 46%, and a median time to progression of 70 days. The study allowed patients to move to cetuximab and platinum-based chemotherapy if they experienced progressive disease on cetuximab monotherapy, but patients who progressed to the combination arm had limited benefit [20]. In 2006, these two landmark studies resulted in the first FDA approval of cetuximab use in combination with RT for LA SCCHN and as monotherapy for platinum-refractory RM SCCHN. 

Panitumumab is a fully humanized IgG2 mAb that inhibits EGFR ligand binding. Several phase II trials conducted in patients with LA or RM SCCHN suggested a benefit of PFS when used in conjunction with chemotherapy [51,52]. Two randomized controlled trials assessed panitumumab in LA SCCHN. The CONCERT-1 trial assessed cisplatin and RT with panitumumab vs. CRT alone in previously untreated LA SCCHN patients. The study did not report an improvement in local control rate or PFS and demonstrated a trend towards worse OS in the panitumumab arm [53]. CONCERT-2 studied panitumumab with radiation vs. CRT alone, but PCR, PFS, and OS favored the CRT arm demonstrating that panitumumab cannot replace cisplatin [54].

The use of zalutumumab in LA SCCHN was assessed in the large phase III randomized trial DAHANCA 19. Patients were randomized to RT or CRT (based on disease stage) with or without zalutumumab. However, there was no improvement in LCR, OS, or disease-specific survival in the zalutumumab arms [55]. Nimotuzumab works similarly to zalutumumab and has been approved in other countries for treatment of SCCHN; however, it has not been approved for use in the United States. Several phase II trials have demonstrated a survival benefit with nimotuzumab and CRT [56,57]. A phase III trial demonstrated improved PFS, LRC, and DFS with the addition of nimotuzumab to concurrent cisplatin CRT in LA disease [58].

Small molecule TKIs are also being studied for use in LA SCCHN and include single targeted agents such as gefitinib and erlotinib and multitargeted TKIs such as lapatinib and afatinib. A single arm phase II study assessed oral gefitinib with concurrent platinum-based CRT in LA SCCHN and reported a one year OS of 87% and a distant metastatic control rate of 98%. However, the study had five treatment-related deaths and overall had high levels of toxicity [59]. As discussed earlier, multitargeted TKIs in high risk SCCHN failed to show additional survival benefits [40,43]. An initial phase I/II study with CRT and erlotinib demonstrated an improved complete response rate (CRR) and OS at 3 years of 72% [60]. However, a phase II trial assessing CRT with or without erlotinib had worse CRR (52% vs. 40%, *p* = 0.08) and no improvement in PFS demonstrating the need for further investigation in this area [61]. Table 1 lists current ongoing or unpublished trials with EGFR inhibition in locally advanced disease.

## 7. Role of EGFR Inhibition in Recurrent Metastatic Disease in SCCHN

Despite the vast number of completed and ongoing studies aiming to target EGFR and other downstream signaling pathways, cetuximab remains the only FDA approved EGFR targeted mAb for the treatment of SCCHN. Prior to the introduction of molecularly targeted agents, first line treatment for RM SCCHN was largely comprised of several cytotoxic agents that were associated with significant morbidity [62]. Eventually, cisplatin was studied in SCCHN and was found to have a favorable response rate (RR) compared to best supportive care (BSC) and methotrexate. The addition of 5-FU to cisplatin improved objective response without improving OS, but due to the tolerable side effect profile became the primary regimen for RM SCCHN [63]. 

The benefits demonstrated by single agent cetuximab fostered studies assessing cetuximab with cisplatin as a first line therapeutic option. The Eastern Cooperative Oncology Group (ECOG) studied cisplatin plus cetuximab vs. cisplatin plus placebo, which demonstrated significant improvement in ORR with the addition of cetuximab (26% vs. 10%), but failed to show significant improvements in OS (9.2 vs. 8.0 months) and PFS (4.2 vs. 2.7 months) [64]. The landmark EXTREME trial randomized 442 patients with RM SCCHN to platinum-based chemotherapy, 5-FU, and cetuximab vs. platinum-5FU and placebo for a maximum of six cycles. There was a statistically significant improvement in prolonged median OS (10.1 vs. 7.4 months), prolonged median PFS (5.6 vs. 3.3 months), and increased RR (20% to 36%) favoring the cetuximab arm [65]. This trial led to the approval of cetuximab in combination with platinum and 5FU-based therapy as first line treatment of recurrent metastatic SCCHN [16]. 

As mentioned above, panitumumab has failed to show significant improvement in LA SCCHN. The phase III trial, SPECTRUM, assessed cisplatin and 5-FU with or without panitumumab in patients with RM SCCHN. The authors reported a statistically significant difference in PFS (5.8 vs. 4.6 months), but the study failed to meet the primary endpoint of OS [66]. The PARTNER trial evaluated whether cisplatin and docetaxel with or without panitumumab could be a potential first line therapy in RM SCCHN. The primary endpoint, PFS, was improved in the panitumumab arm (6.9 vs. 5.5 months, *p* = 0.048), but there was no difference in OS. Additionally, the experimental arm had higher rates of grade 3 and 4a adverse events [51]. This stark contrast from the efficacy demonstrated by cetuximab may be attributed to the lack of ADCC activity secondary to panitumumab’s IgG2 subclass, and may be due to the possible access to cetuximab by some patients [15]. Zalutumumab has shown benefit in PFS but not OS, when compared to BSC in patients with platinum refractory RM SCCHN [67]. There are currently no active trials with zalutumumab. 

In terms of small molecule TKI, several studies with single-target TKIs have been conducted in RM SCCHN as mentioned above. Gefitinib monotherapy had a RR of 10.6%, a DCR of 53%, and a median OS of 8.1 months with a daily dose of 500 mg [68]. However, in a phase III trial comparing gefitinib vs. methotrexate in RM SCCHN, the gefitinib arms failed to improve OS and demonstrated no differences in ORR [69]. Another phase III trial assessing the addition of gefitinib to docetaxel did not improve OS [70]. Erlotinib monotherapy has reported an IRR of 4.3%, a median PFS of 9.6 weeks, and a median OS of 6 months [71]. A randomized phase II study with cisplatin, docetaxel, and erlotinib in patients with RM SCCHN demonstrated improved PFS [72]. 

Multi-targeted TKIs such as afatinib and dacominitib have been studied in RM SCCHN. In the phase III LUX-Head and Neck 1 trial, patients with RM SCCHN refractory to first line platinum-based chemotherapy were randomized to receive oral afatinib vs. intravenous methotrexate. The authors reported improved PFS (2.6 vs. 1.7 months) in the afatinib group vs. the methotrexate group, respectively [73]. Dacomitinib demonstrated a partial response as a monotherapy in platinum progressed RM SCCHN [45,46]. Table 2 lists current active or unpublished trials involving treatment of RM SCCHN.

## 8. Current Applications of EGFR Inhibition in SCCHN in the Era of Immunotherapy

Despite the addition of EGFR targeted therapy, the survival rate of patients with RM SCCHN remains quite low. Since their approval in the treatment of RM SCCHN, immune checkpoint inhibitors (ICIs) are being investigated as an adjunct to EGFR inhibition. The programmed cell death 1 (PD-1) is expressed on activated T cells and programmed cell death ligand 1 (PD-L1) is upregulated on tumor cells possibly by activation of EGFR and PI3K-Akt or JAK/STAT pathways. Targeting the cytotoxic T lymphocyte antigen-4 (CTLA4) immune checkpoint has also been suggested from preclinical data. This axis serves a major role in SCCHN immune evasion [74]. Cancer immunotherapy currently relies on the inhibition of immune checkpoints to hinder the PD-1/PD-L1 axis which allows for recovery of tumor specific immunity of T cells [75,76]. Cancer cells are recognized as foreign antigens by immune cells thus stimulating the adaptive immune system to direct a response against the cancer cells [75]. However, it is well known that cancer cells are able to evade this immune response by preventing recognition or cytotoxic actions of T cells through immune escape mechanisms such as the PD-1/PD-L1 axis [63].

The recent approval of the ICIs, pembrolizumab and nivolumab, for the treatment of the RM SCCHN has changed the therapeutic landscape of this disease. Both are humanized IgG4 mAbs with high affinity for PD-1 receptors, and thereby block the PD-1/PD-L1 axis [63]. Nivolumab was FDA approved after the CHECKMATE-141 trial which compared nivolumab to standard of care (SOC, methotrexate, docetaxel, or cetuximab). The study randomized 362 patients with progressive RM SCCHN, despite them receiving platinum-based therapy and reported significant improvement in median OS (7.5 vs. 5.1 months, *p* = 0.01) and ORR in the nivolumab group (13.3% vs. 5.8%, 95% CI 1.07–5.82 ). Although PFS was shorter in the nivolumab arm (2.0 vs. 2.3 months), the rate of PFS at 6 months was higher in the nivolumab arm (19.7% vs. 9.9%) [77]. These findings were confirmed at a 2-year follow-up analysis [78]. This trial led to the approval of nivolumab for patients with RM SCCHN who failed platinum-based therapy [77]. In post hoc analysis, nivolumab demonstrated significant improvement in 30-month OS in age groups <65 and >65 when compared to SOC [79]. The increased efficacy of nivolumab was unrelated to prior cetuximab use [80]. Additionally, tumor burden reduction occurred in 18 of 60 patients receiving nivolumab after RECIST-defined progression demonstrating possible efficacy in this subset of patients [81]. Nivolumab is also being assessed in combination therapy. CheckMate651 is currently assessing nivolumab and ipilimumab compared to the EXTREME regimen (NCT02741570) and CheckMate714 is currently assessing nivolumab with and without ipilimumab in RM SCCHN (NCT02823574).

The phase1b trial KEYNOTE-012 assessed pembrolizumab in tumors with PD-L1 expression >1% and demonstrated an ORR of 18% in all patients, an ORR of 25% in HPV+ patients, and a median OS of 8 months with a favorable side effect profile. A follow up two-year analysis continued to demonstrate ongoing response in some patients [82,83]. These findings were confirmed in a phase II single-arm study, KEYNOTE-055, which assessed 171 patients with platinum- and cetuximab- refractory RM SCCHN and demonstrated an ORR of 16.4% in all patients, a median OS of 8 months, and a PFS of 2.1 months [84]. To assess whether pembrolizumab monotherapy was comparable to SOC in RM SCCHN, a phase III randomized study, KEYNOTE-040, compared pembrolizumab to investigators’ choice of methotrexate, docetaxel, or cetuximab. The study found median OS in intention-to-treat analysis of 8.4 months vs. 6.9 months in the pembrolizumab and SOC groups, respectively. Additionally, the SOC group had more grade 3 or higher adverse effects. These results suggested that pembrolizumab monotherapy in progressive RM SCCHN could be an alternative to more traditional cytotoxic chemotherapies [85]. However, to characterize the effects of the addition of pembrolizumab to chemotherapy on OS the KEYNOTE-048 trial allocated 882 patients to pembrolizumab monotherapy, pembrolizumab with platinum-based chemotherapy, or chemotherapy with cetuximab as first line treatment. Of the 882 patients, 85% had a PD-L1 combined positive score (CPS) of 1 or more and 43% had CPS of 20 or more, where a higher CPS value signifies increased PD-L1 by tumor cells. The pembrolizumab monotherapy group vs. cetuximab with chemotherapy showed improved OS in patients with CPS of 20 or more (median 14.9 vs. 10.7 months, hazard ratio 0.61, 95% CI 0.45–0.83) and in CPS of 1 or more (median 12.3 vs. 10.3 months, hazard ratio 0.78, 95% CI 0.64–0.96), but was non-inferior in the total population (11.6 vs. 10.7 months). Additionally, pembrolizumab with chemotherapy improved OS vs. cetuximab with chemotherapy (13 vs 10.3 months, hazard ratio 0.77, 95% CI 0.63–0.93). These findings suggest pembrolizumab plus platinum based chemotherapy is appropriate for first line RM SCCHN, and pembrolizumab monotherapy can be used for PD-L1 positive RM disease [86]. Currently, there are several ongoing studies assessing combination immunotherapy, immunotherapy with RT, and immunotherapy with chemotherapy for possible synergistic effects [75]. 

Monotherapy with the PD-L1 inhibitor durvalumab was investigated in the HAWK trial and demonstrated antitumor activity with an acceptable safety profile in patients with >25% PD-L1 expressing tumor cells [87]. The phase II CONDOR study demonstrated a manageable safety profile with durvalumab, tremelimumab (CTLA-4 inhibitor), and combination durvalumab–tremelimumab. It also demonstrated clinical benefit in both durvalumab monotherapy and combination arms in RM SCCHN in an ongoing phase III trial [88]. These trials suggest durvalumab demonstrates efficacy regardless of PD-L1 expression. In contrast to these trials, the EAGLE study did not demonstrate improvement in OS with either durvalumab monotherapy or durvalumab plus tremelimumab compared to SOC in second-line RM SCCHN [89]. Of note, these negative results may be secondary to the high rate of immunotherapy received by the SOC arm [90].

In terms of LA disease, a phase Ib study with 59 patients assessed pembrolizumab with weekly cisplatin-based CRT and demonstrated a favorable toxicity profile, with an end of treatment complete response of 85.3% and 78.3% based on imaging in HPV positive and negative groups, respectively [91]. Currently, a phase III trial KEYNOTE-412 is ongoing and is assessing pembrolizumab with CRT in LA SCCHN (NCT03040999) [92]. In addition, a trial assessing dual ICI blockade with RT in LA SCCHN is actively recruiting patients (NCT03426657). 

## 9. Dual EGFR Inhibition and Immunotherapy

Both EGFR mAbs and ICIs have individually demonstrated significant success in the treatment of advanced SCCHN. Due to this success, there is significant interest in assessing treatment efficacy with dual inhibition. EGFR inhibition has a large impact on the tumor microenvironment through activation of ADCC via NK cells, promoting cross-talk between NK cells and dendritic presenting cells (DC), and priming cytotoxic T cells [93]. However, these immune related mechanisms lead to negative feedback loops which may decrease the efficacy of EGFR-targeted therapy. For example, cetuximab’s ADCC activity can stimulate IFN-y secretion from NK cells, improving NK and DC cross-talk, but it also induces PD-L1 expression and therefore inhibits active T and NK cells and assists in tumor immune escape [93,94]. This suggests that the simultaneous use of cetuximab and ICIs may have synergistic effects that can improve patient outcomes. 

To assess these synergistic effects a phase I/II study evaluated the combination of cetuximab and nivolumab in RM SCCHN and found it was well-tolerated, but it did not improve OS at one year in this heavily pre-treated population. However, cetuximab and nivolumab combination therapy did have a more favorable PFS trends in patients without prior ICI therapy [95]. Currently, a study assessing pembrolizumab and cetuximab in RM SCCHN (NCT03082534) is actively recruiting patients [96]. For LA SCCHN, a phase III trial assessing avelumab–cetuximab-RT, RT-cisplatin, vs. RT–cetuximab in 41 patients was found to have an acceptable toxicity profile and is awaiting further results [97]. Another study assessing combination therapy with nivolumab and EGFR therapy in cisplatin-ineligible patients has been completed and is awaiting results [9].

Novel approaches beyond the combination of ICI and cetuximab will be crucial. NK cells play a significant role in the immune related mechanisms of cetuximab action and cannot be overlooked. NK cells can be suppressed in SCCHN through inhibitory ligands and targeting these inhibitory NK cell receptors may serve as another area of therapy [98]. One such receptor is the NKG2A, which can be targeted with the mAb inhibitor monalizumab. A phase II trial studied the combination of monalizumab and cetuximab in RM SCCHN and demonstrated partial responses in 8 of 26 patients and stable disease in 14 of 26 patients [99]. Another potential therapeutic involves motolimod, a toll-like receptor 8 agonist, which may interact with the innate and adaptive immune response. It has been studied in combination with the EXTREME regimen and has a favorable toxicity profile; however, it did not improve PFS or OS in RM SCCHN [100]. There are currently several ongoing studies with various combination therapies assessing response rates in SCCHN.

## 10. Conclusions

Effective therapies for locally advanced and RM SCCHN remain elusive. Unfortunately, even with the extensive research involving EGFR inhibition as well as immunotherapy, SCCHN remains a very challenging disease with an overall poor prognosis. The more recent development of ICIs such as pembrolizumab gives hope for new effective treatment strategies that have the potential to improve survival while offering acceptable toxicity profiles. However, there is minimal data on combination therapy with EGFR inhibition and PD-1/PD-L1 blockade. Given the in vitro data and limited early phase trials, these combinations deserve to be pursued with the goal of providing patients with decreased treatment toxicity, improved quality of life, and a significant improvement in overall survival.

## Figures and Tables

**Table 1:** Current ongoing trials for EGFR inhibition in definitive disease.

Study (NCT/Phase)	Therapeutic Arms	Primary Endpoint
NCT00865098 (Phase II)	Cetuximab + RT	Completion rate
NCT01154920 (Phase II)	Group A: Cetuximab + paclitaxel + carboplatin Group B: Cetuximab + docetaxel + cisplatin + 5-FU	Progression free survival
NCT01515137 (Phase I)	Group A: Erlotinib Group B: Erlotinib + sulindac Group C: Placebo	Change in Ki67 proliferative index
NCT00079053 (Phase I)	Adjuvant erlotinib	Toxicity/dose
NCT04091867 (Phase I)	sEphB4-HAS + cetuximab + RT	Dose limiting toxicity
NCT01737008 (Phase I)	Dacomitinib + RT +/− cisplatin	Dose limiting toxicity
NCT00304278 (Phase II)	Erlotinib + RT + cisplatin	Complete and partial response rate
NCT00371566 (Phase II)	CRT +/− lapatinib	Change in apoptotic index
NCT01592721 (Phase I/II)	Cetuximab + EGFR antisense DNA + RT	Safety, efficacy
NCT01218048 (Phase II)	Neo-adjuvant cetuximab + surgery + CRT	Biomarker (NK cell activation)
NCT00055770 (Phase I/II)	Erlotinib + docetaxel	Dose limiting toxicity
NCT00720304 (Phase II)	Erlotinib + docetaxel + RT	Time to progression
NCT02537223 (Phase I)	BYL719 + cisplatin + IMRT	Treatment related side effects
NCT02051751 (Phase Ib)	BYL719 + paclitaxel	Dose limiting toxicity
NCT03051906 (Phase I/II)	Cetuximab + durvalumab + IMRT	Progression free survival
NCT02979977 (Phase II)	Afatinib + cetuximab	Objective response rate

Key: Radiation therapy (RT), 5-Fluorouracil (5-FU), sEphB4-HAS (EphrinB2 inhibitor), chemoradiotherapy (CRT), BYL719 (alpha-specific PI3K inhibitor), intensity-modulated radiation therapy (IMRT).

**Table 2:** Current ongoing trials for EGFR inhibition in recurrent/metastatic disease.

Study (NCT/Phase)	Therapeutic Arms	Primary Endpoint
NCT02054442 (Phase Ib/II)	Group A: Cetuximab + methotrexate Group B: Methotrexate	Dose limiting toxicity, PFS
NCT02057107 (Phase II)	Group A: SBRT + cetuximab + docetaxel followed by cetuximab + docetaxel Group B: SBRT + cetuximab followed by cetuximab	PFS
NCT04375384 (Phase II)	Cetuximab after immunotherapy	Objective response rate
NCT01334177 (Phase I)	VTX-2337 + cetuximab	Safety, tolerability, and dose limiting toxicity
NCT04428151 (Phase II)	Group A: Pembrolizumab + lenvatinib Group B: SOC therapy Group C: Lenvatinib	Objective response rate
NCT02268695 (Phase II)	Group A: Cisplatin + 5-FU + cetuximab Group B: Cisplatin + docetaxel + cetuximab	Overall survival
NCT04199104 (Phase III)	Pembrolizumab +/− lenvatinib	Objective response rate
NCT00098631 (Phase II)	Lapatinib	ORR, PFS, and toxicity
NCT01316757 (Phase II)	Cetuximab + paclitaxel + carboplatin + erlotinib	Objective response rate
NCT01064479 (Phase II)	Carboplatin/cisplatin + docetaxel + erlotinib	Progression free survival
NCT01577173 (Phase II)	MEHD7945A vs. cetuximab	Progression free survival
NCT02277197 (Phase I)	Ficlatuzumab + cetuximab	dosing
NCT04590963 (Phase III)	Monalizumab + cetuximab	Overall survival
NCT03422536 (Phase II)	Ficlatuzumab +/− cetuximab	Progression free survival
NCT01044433 (Phase II)	Lapatinib + capecitabine	Overall survival
NCT03109158 (Phase I/II)	NC-6004 + cetuximab + 5-FU	Dose limiting toxicity
NCT03646461 (Phase II)	Group A: Ibrutinib + cetuximab Group B: Ibrutinib + nivolumab	Overall response rate
NCT00114283 (Phase II)	Lapatinib	Objective response rate
NCT03370276 (Phase I/II)	Nivolumab + cetuximab	Recommended phase II dose
NCT03695510 (Phase II)	Afatinib + pembrolizumab	Objective response rate
NCT02643550 (Phase I/II)	Monalizumab + cetuximab	Safety, ORR
NCT03082534 (Phase II)	Cetuximab + pembrolizumab	Objective response rate

Key: Stereotactic body radiation therapy (SBRT), VTX-2337 (TLR8 agonist), Standard of Care (SOC), 5-Fluorouracil (5-FU), MEHD7945A (dual EGFR/HER3 inhibitor), NC-6004 (novel cisplatin nanoparticle).
